# Structural Characterization of Lignin and Lignin-Carbohydrate Complex (LCC) from Ginkgo Shells (*Ginkgo biloba* L.) by Comprehensive NMR Spectroscopy

**DOI:** 10.3390/polym10070736

**Published:** 2018-07-04

**Authors:** Bo Jiang, Yu Zhang, Tianyu Guo, Huifang Zhao, Yongcan Jin

**Affiliations:** 1 Jiangsu Co-Innovation Center of Efficient Processing and Utilization of Forest Resources, Nanjing Forestry University, Nanjing 210037, China; 111501206@njfu.edu.cn (B.J.); 121502225@njfu.edu.cn (Y.Z.); gty@njfu.edu.cn (T.G.); nuzhenzi_1984@163.com (H.Z.); 2 Institute of Botany, Jiangsu Province and the Chinese Academy of Sciences, Nanjing 210014, China

**Keywords:** ginkgo shells, Lignin, lignin-carbohydrate complex, structure, NMR

## Abstract

Lignin and lignin-carbohydrate complexes are important polymers for lignocellulosic biorefinery and functional materials, but those in ginkgo shells are not effectively analyzed and exploited. Based on this background, milled wood lignins (MWL_ML_ and MWL_FZ_) and lignin-carbohydrate complexes (LCC_ML_ and LCC_FZ_) were isolated from the shells of *Ginkgo biloba* L. cv. Damaling (ML) and *Ginkgo biloba* L. cv. Dafozhi (FZ) correspondingly, and were structurally characterized by comprehensive NMR spectroscopy. The results showed that ginkgo shells exhibited higher lignin (42%) and xylan (20%) content than general softwood species. Isolated MWLs were rich in guaiacyl units with the presence of ferulates and *p*-coumarates, and the molecular formula was C_9_H_7.93_O_2.73_(OCH_3_)_0.81_ and C_9_H_7.87_O_2.76_(OCH_3_)_0.88_ for MWL_ML_ and MWL_FZ_, respectively. Phenolic hydroxyl of MWL_ML_ (1.38 mmol/g) and MWL_FZ_ (1.23 mmol/g) in ginkgo shells was much less than that in general softwoods, suggesting a higher etherification and condensation degree of ginkgo shells lignin, and *β*-5′, *α*-O-4′, and 4-O-5′ bonds were the main condensed structures. *O*-acetylated *β*-d-xylopyranoside and *β*-d-mannopyranoside were the main polysaccharides associated with lignin, and the acetyl groups frequently acylate the C_2_ and C_3_ positions. LCC_ML_ had more phenyl glycoside (0.035/Ar) and less *γ*-ester (0.026/Ar) linkages than LCC_FZ_.

## 1. Introduction

Lignocellulosic biomass is one of the economically viable and environmentally sustainable feedstocks for replacing fossil resources. It is mainly composed of three biopolymers: cellulose (40–60%), hemicelluloses (10–40%), and lignin (15–30%) [1]. Lignin is a complex macromolecule mainly synthesized from three *p*-hydroxycinnamyl alcohols (*p*-coumary, conifery, and sinaply) which give rise to *p*-hydroxyphenyl, guaiacyl, and syringyl units with a variety of interunit linkages (*β*-O-4′, *α*-O-4′, *β*-5′, *β*-1′, 5-5′, 4-O-5′, *β*-*β*′, and so on). These structural units and linkages of lignin give plant cell walls many physicochemical properties and biological activities such as their rigidity and pathogen defense [2], and it is an important biopolymer for valorization [3,4]. For example, the phenolic groups in the lignin structure can be used to produce phenol formaldehyde resins [5]. Lignin with a low molecular weight and high quantity of phenolic hydroxyl groups positively contributes to the mechanical properties of a polyethylene-lignin composite [6]. Lignosulfonate, a water-soluble derivative of lignin, can utilize the redox chemistry of quinone to store energy [7]. These results suggest that the physicochemical properties and active groups of lignin play a crucial role in the production of materials. Therefore, the structural analysis of lignin is the prerequisite for its modification and functionalization.

Although extensively investigated, the complex and irregular structure of lignin is still not completely elucidated. Wet chemistry techniques such as nitrobenzene oxidation [8], ozonation [9,10], derivatization followed by reductive cleavage [11,12], and so on, can provide precise and specific structural information on lignin. However, each wet chemistry technique is not able to offer a visual picture of the entire lignin structure, and the experimental process of some chemical degradation methods is cumbersome, tedious, and even toxic.

Various spectroscopic methods such as infrared, ultraviolet-visible, Raman spectroscopy, and nuclear magnetic resonance (NMR), have been applied to analyze the lignin structure. NMR is a power tool able to probe the structure of lignin, even the lignin-carbohydrate complex (LCC). The advantage of NMR over other spectroscopic techniques is that NMR has a much higher resolution, enabling a larger amount of information on lignin structural units and side-chain linkages to be obtained [13]. For example, ^1^H NMR is a common spectroscopic technique providing information on hydrogen protons in lignin, especially the –OCH_3_ content [14]. ^31^P NMR has been employed to detect and quantify the aliphatic, phenolic hydroxyl, and carboxy groups [15]. ^13^C NMR and two-dimensional heteronuclear single quantum coherence (2D HSQC) NMR have also been developed to analyze lignin structure and LCC linkages [13,16,17]. In addition, although quantitative ^13^C and 2D HSQC NMR used solely is not efficient for the quantification of LCC linkages due to the enrichment of associated carbohydrates with lignin, the combination of ^13^C NMR of lignin and 2D HSQC NMR of LCC, proposed by Zhang and Gellerstedt, is considered to be an effective method [18,19]. This approach uses an appropriate resonance in a quantitative ^13^C NMR spectrum as an internal reference to convert relative integration values obtained from the corresponding 2D HSQC NMR spectrum to the absolute values.

*Ginkgo biloba* is a Mesozoic tree species, and research on *Ginkgo biloba* mainly focuses on the ginkgo leaf, fruit, and bark. However, the ginkgo shells as residues are not effectively exploited. Although ginkgo shells are annual tissues, the rigidity of ginkgo shells is at a high level, and the occurrence of lignin is considered to be the main reason for this, as Shen et al. pointed out that the lignin content in the shells of Ginkgo (*Ginkgo biloba*), Macadamia nut (*Macadamia ternifolia F. Muell.*), Pine nut (*Arachis hypogaea Linn.*), and Pecan (*Carya illinoinensis Wangenh. Koch*) could be up to 37.87%, 42.45%, 43.66%, and 39.06, respectively. That of Almond (*Prunus amygdalus*), Chestnut (*Castanea mollissima Blume*), Pumpkin seed (*Cucurbita moschata*), and Lotus seed (*Nelumbo nucifera Gaertn.*) is only 28.12%, 18.15%, 15.12%, and 10.10%, respectively [20]. In addition, the ginkgo shells have high antioxidant activity, protecting the fruit in ginkgo shells from pathogens. These phenomena indicate that the biosynthesis of lignin in ginkgo shells may be different from that in general softwoods, resulting in different chemical compositions, physicochemical properties, and biological activities. These structural properties have important effects on biorefinery and materials production of lignin. Based on this background, and for the application of ginkgo shells beyond their current roles, MWL and LCC preparations were isolated from ginkgo shells (*Ginkgo biloba* L. cv. Damaling and *Ginkgo biloba* L. cv. Dafozhi) in this work and structurally characterized by comprehensive NMR spectroscopy (^1^H, ^13^C, 2D HSQC, and ^31^P) to identify and quantify the lignin structure and LCC linkages.

## 2. Materials and Methods

### 2.1. Materials

The shells of two ginkgo species, *Ginkgo biloba* L. cv. Damaling (ML) and *Ginkgo biloba* L. cv. Dafozhi (FZ), were collected from Taizhou, Jiangsu province, China (subtropical humid climate zone). Air-dried ginkgo shells were sealed in plastic bags and stored in a refrigerator at 4 °C before use. The chemicals used in this work were all analytical or reagent grade and used as received without further purification.

### 2.2. Isolation of MWLs and LCCs

**Figure 1:**
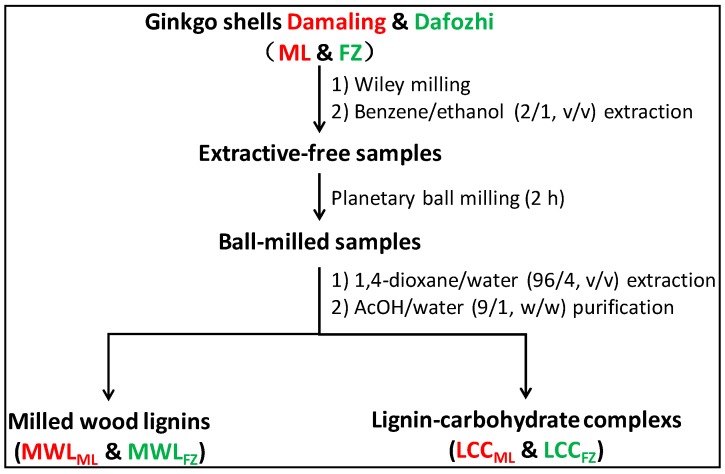
Isolation procedure of MWLs and LCCs from ginkgo shells.

Milled wood lignin (MWL) was isolated according to the method described by Björkman [21], and the isolation procedure of lignin and LCC preparations from ginkgo shells is illustrated in Figure 1. The air-dried ginkgo shells were ground in a Wiley mill. Particles between 40 mesh (0.425 mm) and 80 mesh (0.180 mm) were extracted with ethanol/benzene (1:2, *v*/*v*) in a Soxhlet extractor for 12 h to obtain extractive-free samples. The extractive-free shell meals were milled in a planetary ball mill (Fritsch GMBH, Pulverisette 7 premium line, Idar-Oberstein, Germany) at a frequency of 10 Hz for 2 h without solvent. Two 80 mL silicon nitride bowls, with 4 g ginkgo shell meals in each bowl, were filled with 25 zirconium dioxide balls (1 cm diameter). The milling was conducted at room temperature, and 15 min intervals were provided between every 3 min of milling to prevent overheating.

The ball-milled samples were suspended in 1,4-dioxane (96%, *v*/*v*) with a liquid-to-solid ratio of 15 (mL/g) at room temperature for 24 h. The extraction procedure was conducted in the dark and under a nitrogen atmosphere. The mixture was centrifuged and washed using 96% 1,4-dioxane until the filtrate was clear. Such operations were repeated thrice. The extractive supernatants were combined and the solvent was recycled by vacuum evaporation. The crude lignin was dissolved in acetic acid (90%, *w*/*w*), and the soluble fraction was slowly introduced into deionized water. The supernatants were vacuum evaporated and washed to obtain LCCs (LCC_ML_ and LCC_FZ_). The precipitates were washed with deionized water to obtain lignin preparations (MWL_ML_ and MWL_FZ_). No further purification was performed for the preservation of the structural features of the lignin preparations.

### 2.3. Analytical Methods

Chemical components of ginkgo shells, MWLs, and LCCs were analyzed according to our previous work [22]. An elemental analyzer (2400 II, Waltham, MA, USA) was used to measure the content of carbon (C), hydrogen (H), nitrogen (N), and sulfur (S) elements of MWLs, and the amount of oxygen (O) element was calculated by the difference. The ^13^C NMR of MWLs and 2D HSQC NMR of MWLs and LCCs were determined according to the method described by Huang et al. [23]. Acetylated MWLs were used for the determination of ^1^H NMR spectra and molecular weight distribution [24].

Quantitative ^31^P NMR was used for the determination of hydroxyl content in MWLs. A total of 40 mg of samples was dissolved in 500 µL of an anhydrous pyridine/CDCl_3_ mixture (1.6/1, *v*/*v*). Then, 200 µL of an endo-*N*-hydroxy-5-norbornene-2,3-dicarboximide (9.23 mg/mL, internal standard) and 50 µL of chromium (III) acetylacetonate (5.6 mg/mL, relaxation reagent), prepared using the anhydrous pyridine/CDCl_3_, were added. The lignin solution was reacted with 100 µL of phosphitylating reagent (2-chloro-4,4,5,5-tetramethyl-1,2,3-dioxaphosphplane) for 15 min and then transferred into an NMR tube for ^31^P NMR analysis. All NMR spectra of MWLs and LCCs were acquired on a Bruker AVANCE III 600 MHz spectrometer (Bruker, Biospin, Switzerland) equipped with a 5 mm BBO probe using an inverse gated proton decoupling sequence.

For comparison, the wet chemistry, alkaline nitrobenzene oxidation, was applied to the extractive-free ginkgo shells (40–80 mesh) and LCCs according to the procedure reported by Chen [8]. Briefly, 10 mg of each sample was reacted with 4 mL 2 mol/L sodium hydroxide and 0.25 mL nitrobenzene in a stainless steel bomb at 170 °C for 2 h. Then, the bomb was cooled in cold water immediately and 1 mL of 0.1 mol/L sodium hydroxide solution containing 3-ethoxy-4-hydroxybenzaldehyde (0.3 g/L) was used as the internal standard. The mixture was extracted three times with dichloromethane. The aqueous phase was acidified with 4 mol/L HCl to pH 1 and extracted twice with dichloromethane and once with ethyl ether. The combined organic phase was extracted with 20 mL deionized water and the organic phase was dried by anhydrous sodium sulfate overnight. After removing the insoluble inorganic materials by filtration, the solution was evaporated to dryness and silylated using *N*,*O*-bis(trimethylsilyl) acetamide at 100 °C for 10 min. The silylated samples were analyzed by gas chromatography (Plus 2010) equipped with a flame ionization detector and SH-Rtx-5 column (Shimazu Co., Kyoto, Japan).

## 3. Results and Discussion

### 3.1. Chemical and Elemental Composition

The main chemical components of ginkgo shells (ML and FZ), MWLs (MWL_ML_ and MWL_FL_), and LCCs (LCC_ML_ and LCC_FZ_) are given in Table 1. The amount of lignin (42%) and xylan (20%) in ginkgo shells was much higher than that of lignin (25–30%) and xylan (5–10%) in general softwood species [25], so it is reasonable to deduce that the lignin-xylan complex is one of the main covalent forms of LCC in ginkgo shells. The high lignin and xylan content may be one of the reasons that the rigidity of ginkgo shells is very high. The content of glucan in ginkgo shells is only about 20%, which means that the amount of cellulose in ginkgo shells is much less than that in general softwoods. Research has pointed out that *Ginkgo biloba* woods contain about 41% cellulose, 33% lignin, and 26% hemicelluloses, and the predominant polysaccharides in hemicelluloses are glucomannan and methylglucuronoxylan [26]. The main chemical composition of *Ginkgo biloba* leaves is terpene trilactones, flavonoids, and other trace constitutes [27]. These results suggested that the chemical composition of ginkgo shells is rather different from that of ginkgo woods and leaves.

The yield of isolated MWL_ML_ and MWL_FZ_, on the basis of total lignin in ginkgo shells, was only 17.5% and 15.3%, respectively. Due to the high rigidity of ginkgo shells and the fact that the lignin is mainly deposited in secondary cell walls [2], the ball milling degree in this work (2 h) may not completely destroy the cell walls, resulting in low-level lignin being extracted out. However, the purity of both MWLs was over 90%, meeting the need of structural analysis. The main polysaccharides in LCCs were glucan and xylan, and expectedly, the LCCs had a higher acid soluble lignin content than raw materials and MWLs owing to the low molecular weight of lignin in LCCs.

MWL_ML_ and MWL_FZ_ exhibited a similar elemental composition, as shown in Table 2. The N and S elements were present in MWLs besides the predominant C, H, and O elements. During lignification, the bio-enzymes such as phenylalanine ammonia-lyase, cinnamate 4-hydroxylase, 4-coumarate: CoA ligase, and *p*-coumarate 3-hydroxylase play an important role in the formation of lignin units and interunit linkages [28]. Ginkgo shells used in this work were extracted by benzene-ethanol solvent and no specific step was carried out to remove protein. Therefore, the N and S elements in MWLs could be mainly derived from protein.

### 3.2. Molecular Weight Distribution

Technically, the molecular weight distributions are dependent on the isolation and detection methods. The values of the weight-average (M_w_), number-average molecular weights (M_n_), and the polydispersity (M_w_/M_n_) of MWL_ML_ and MWL_FZ_ are given in Table 2. MWL_ML_ and MWL_FZ_ exhibited similar M_w_, with a relative value of 12,130 Da and 11,550 Da, respectively. Both MWLs show a broad molecular weight distribution with the polydispersity data of 3.19 and 2.94 for MWL_ML_ and MWL_FZ_, respectively.

### 3.3. ^1^H NMR Spectra of MWLs

**Figure 2:**
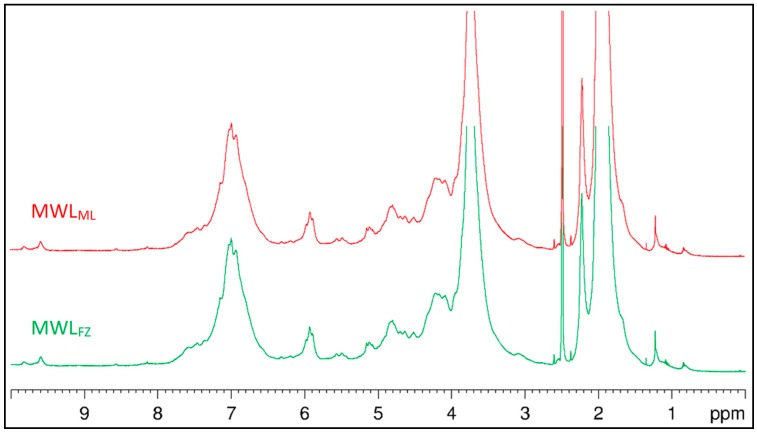
The ^1^H NMR spectra of MWL_ML_ and MWL_FZ_.

The ^1^H NMR spectra of acetylated MWL_ML_ and MWL_FZ_ are presented in Figure 2. Only small differences were found between MWL_ML_ and MWL_FZ_. The broad signal around 7.3–7.6 ppm confirmed the presence of the *p*-coumarate-type structure, C*_α_* = O groups, and *p*-hydroxyphenyl (H) units in both MWLs [29]. Signals of H*_α_* from *β*-O-4′ and *β*-5′interunit linkages could be found at 5.9 and 5.5 ppm, respectively, and the signals’ intensity implied that *β*-O-4′ were the prominent linkages. The proton signals from *β*-*β*′ linkages were not evident compared with that from *β*-5′, indicating that *β*-5′ bonds may be the main condensed structure. –OCH_3_ is an important functional group of lignin, which accounted for about 13.3% and 14.3% of MWL_ML_ and MWL_FZ_, respectively, according to the calculation methods described by Mousavioun and Doherty [14]. The analysis of elements, molecular weight, and –OCH_3_ groups gives the MWL_ML_ and MWL_FZ_ molecular formula C_9_H_7.93_O_2.73_(OCH_3_)_0.81_ and C_9_H_7.87_O_2.76_(OCH_3_)_0.88_, respectively, which is important information on lignin structure for biorefinery and chemical modification.

### 3.4. 2D HSQC NMR Spectra of MWLs

**Figure 3:**
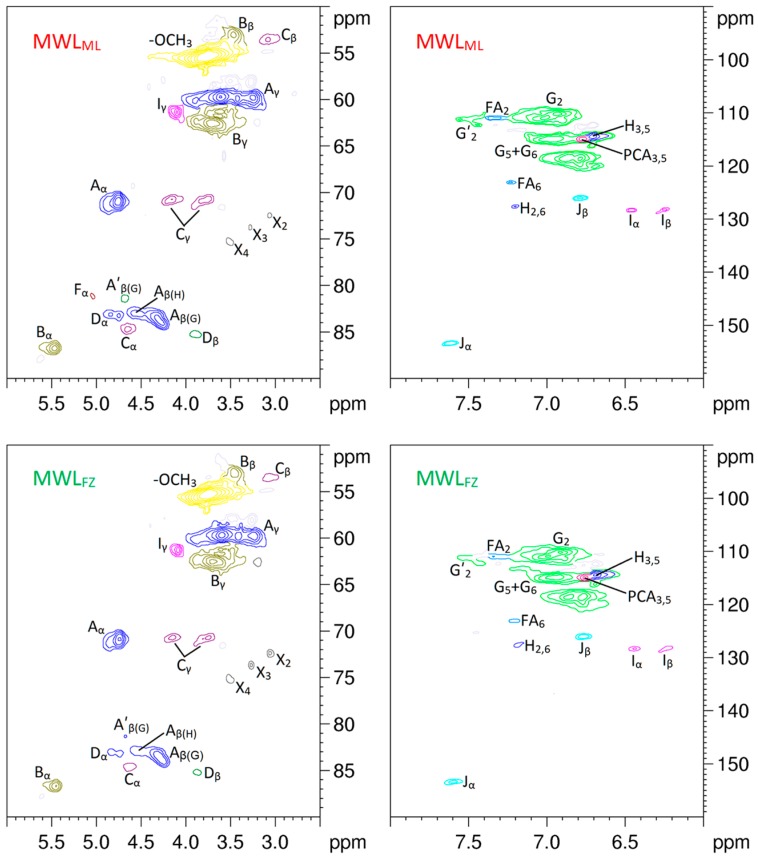
Side-chain (*δ*_C_/*δ*_H_ 90–50/6.0–2.5) and aromatic (*δ*_C_/*δ*_H_ 160–90/8.0–6.0) regions of ginkgo shell MWLs in the 2D HSQC NMR spectra.

**Figure 4:**
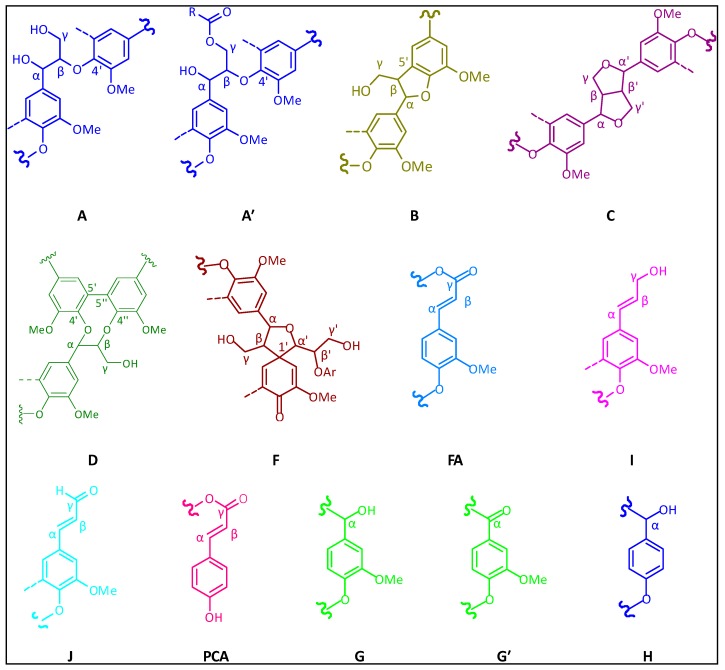
Main substructures in MWL_ML_ and MWL_FZ_: (**A**) *β*-O-4′ linkages with a free –OH at the *γ*-carbon; (**A′**) *β*-O-4′ linkages with acetylated and/or *p*-hydroxybenzoated –OH at the *γ*-carbon; (**B**) phenylcoumaran substructures formed by *β*-5′ and *α*-O-4′ linkages; (**C**) resinol substructures formed by *β*-*β*′, *α*-O-*γ*′, and *γ*-O-*α*′ linkages; (**D**) dibenzodioxocin substructures formed by *β*-O-4′ and *α*-O-4′ linkages; (**F**) spirodienone substructures formed by *β*-1′ and *α*-O-*α*′ linkages; (**FA**) ferulate substructures; (**I**) cinnamyl alcohol end-groups; (**J**) cinnamyl aldehyde end-groups; (**PCA**) *p*-coumarate substructures; (**G**) guaiacyl units; (**G′**) oxidized guaiacyl units with a *α*-ketone; (**H**) *p*-hydroxyphenyl units.

HSQC NMR is an effective and powerful tool used to probe the structure of lignin and its derivatives, and it can determine specific carbon functionalities that are unable to be identified in ^13^C and ^1^H spectra. The side-chain (*δ*_C_/*δ*_H_ 90–50/6.0–2.5) and aromatic (*δ*_C_/*δ*_H_ 160–90/8.0–6.0) regions of the HSQC NMR spectra of MWL_ML_ and MWL_FZ_ are shown in Figure 3, and the structure of the main lignin substructures are depicted in Figure 4. The cross-signals related to the structural units and linkages in the spectra were assigned according to the published papers [30,31,32,33]. The side-chain regions of MWL_ML_ and MWL_FZ_ in the HSQC NMR spectra were similar. Correlation peaks from methoxyl and *β*-O-4′ substructures (A, A′) were the most prominent in the HSQC spectra of MWL_ML_ and MWL_FZ_. The signals for the C*_β_*-H*_β_* correlations of *γ*-acylated *β*-O-4′ substructures (A′) linked to guaiacyl (G) units (A′*_β_*_(G)_) were clearly observed at *δ*_C_/*δ*_H_ 81.6/4.67, indicating a high acylation extent of G-lignin units in ginkgo shells. Correlation signals from *α*,*β*-diaryl ether linkages were not detected in both spectra of MWL_ML_ and MWL_FZ_, indicating that *α*,*β*-diaryl ether was undetectable or present at a very low level in ginkgo shells, although they are easily detected in some herbaceous lignin preparations [34,35]. Other substructures were also clearly visible in the HSQC NMR spectra of the MWLs, including signals for phenylcoumarans (B), resinols (C), and dibenzodioxocins (D), and the small contour revealed that the amount of these substructures is low. Additionally, the minor signal from the C*_α_*-H*_α_* correlation of spirodienones (F) was observed in the HSQC NMR spectrum of MWL_ML_, but was absent in that of MWL_FZ_. It indicated that MWL_ML_ has a higher content of *β*-1′ and *α*-O-*α*′ linkages than MWL_FZ_. Polysaccharide signals, dominated by hemicellulose correlations, were observed in the side-chain regions of the 2D HSQC spectra of MWL_ML_ and MWL_FZ_. The cross-peak signals of X_2_ (*δ*_C_/*δ*_H_ 72.6/3.05), X_3_ (*δ*_C_/*δ*_H_ 73.8/3.25), and X_4_ (*δ*_C_/*δ*_H_ 75.4/3.53) were assigned to the C_2_-H_2_, C_3_-H_3_, and C_4_-H_4_ correlations of *β*-D-xylopyranoside. These signals indicated that xylan was hardly removed from the lignin structure and the covalent bonds were present between them. It was in line with the chemical components analysis (Table 1) which demonstrated that the content of xylan was higher than that of other polysaccharides in MWLs.

The main signals in the aromatic regions of the 2D HSQC NMR spectra of MWLs corresponded to the G and H units. Prominent signals assigned to *p*-coumarate (PCA) and ferulate (FA) structures were also observed, although they are hardly detected in general softwood lignin. The C_2_-H_2_ and C_6_-H_6_ correlations of FA were observed at *δ*_C_/*δ*_H_ 111.1/7.34 and 123.3/7.20, respectively. Only the C_3,5_-H_3,5_ correlations from PCA were detected at *δ*_C_/*δ*_H_ 115.5/6.76, and the C_2,6_-H_2,6_, C*_α_*-H*_α_*, and C*_β_*-H*_β_* correlations were hardly detected in the HSQC spectra. The results revealed that the degree of reduction and the biosynthesis pathway of FA in ginkgo shells may be different from that of PCA. During lignification, the first step in the reduction of FA is the activation of the carboxyl group via the formation of a CoA ester. FA is converted to feruloyl adenylate, which is subsequently converted to feruloyl-caffeate by CoA. Similarly, hydroxycinnamoyl-CoA is reduced to the corresponding aldehydes by *p*-hydroxycinnamoyl-CoA reductase. The reactions are catalyzed by cinnamate:CoA ligase, which is distributed in various higher plants [36]. Although PCA and FA are effective substrates in the reactions, FA provides the initiation sites from which the lignification event in the cell wall begins and PCA is present via lignification using monolignol conjugation [37]. Therefore, the biosynthesis pathway of FA is different from that of PCA, resulting in different C-H correlations in the 2D HSQC spectra. Other signals in the aromatic regions of the HSQC NMR spectra of MWLs are from the unsaturated side-chains of cinnamyl alcohol end-groups and cinnamaldehyde end-groups. The signal of C*_α_*-H*_α_* and C*_β_*-H*_β_* correlations from cinnamyl alcohol end-groups was detected at *δ*_C_/*δ*_H_ 128.3/6.44 and 128.3/6.23, respectively, and that from cinnamaldehyde end-groups was detected at 153.3/7.59 and 126.1/6.76, respectively.

### 3.5. 2D HSQC NMR Spectra of LCCs

**Figure 5:**
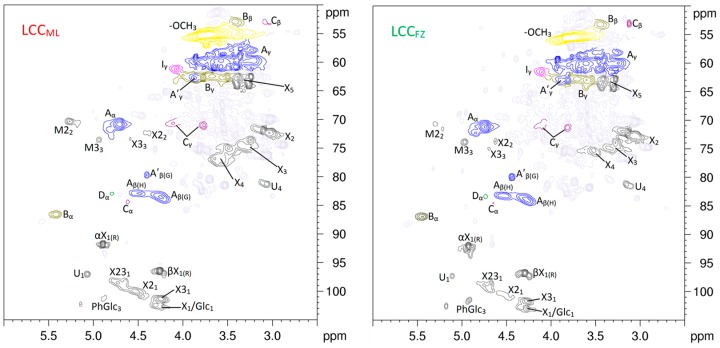
Carbohydrate anomeric regions (*δ*_C_/*δ*_H_ 105–50/6.0–2.5) of LCC_ML_ and LCC_FZ_ in the 2D HSQC NMR spectra. M and X are *β*-d-mannopyranoside and *β*-d-xylopyranoside units, correspondingly. M_2_ and M_3_ are *β*-d-mannopyranoside units *O*-acetylated at C_2_ and C_3_ positions, correspondingly. X2, X3, and X23 are *β*-d-xylopyranoside units *O*-acetylated at C_2_, C_3_, and both positions, correspondingly. *α*X and *β*X are *α*- and *β*-reducing end carbohydrate units, correspondingly.

Almost all wood lignin is associated with polysaccharides, although less LCC exists in the plant. LCC limits the separation of lignin and carbohydrates and the bioconversion in biorefining. Therefore, in view of theory and practice, it is vitally important to understand the native LCC structure in the lignocellulosic biomass. As shown in Figure 5, various signals from the associated carbohydrates could be found in the HSQC spectra of LCCs. Assignment of these polysaccharide signals is listed in Table 3. The signals of *β*-d-xylopyranoside units (X_2_, X_3_, and X_4_) detected in MWLs were also detected in LCCs. The C_2_-H_2_ correlations from 2-*O*-acetyl-*β*-d-xylopyranoside units (X2_2_) and C_3_-H_3_ correlations from 3-*O*-acetyl-*β*-d-xylopyranoside (X3_3_) units were clearly observed at *δ*_C_/*δ*_H_ 72.8/4.43 and 73.9/4.61, respectively. Anomeric correlations from the reducing end of (1→4)-*α*-d-xylopyranside (*α*X_1_) and (1→4)-*β*-d-xylopyranside (*β*X_1_) units were found at *δ*_C_/*δ*_H_ 91.9/4.89 and 96.8/4.26, respectively. The C_1_-H_1_ correlations of 2-*O*-acetyl-*β*-d-xylopyranoside (X2_1_), 3-*O*-acetyl-*β*-d-xylopyranoside (X3_1_), and 2, 3-*O*-acetyl-*β*-d-xylopyranoside (X23_1_) were observed at *δ*_C_/*δ*_H_ 99.8/4.52, 101.3/4.28, and 98.3/4.72, respectively. In addition, the C_2_-H_2_ correlations from 2-*O*-acetyl-*β*-d-mannopyranoside (M2_2_) and 3-*O*-acetyl-*β*-d-mannopyranoside (M2_3_) were detected at *δ*_C_/*δ*_H_ 70.3/5.27 and 73.6/4.93, respectively. These results suggested that the *O*-acetyl-*β*-d-xylopyranoside and *β*-d-mannopyranoside were the main polysaccharides associated with lignin, and the acetyl groups frequently acylate the C_2_ and C_3_ positions. You et al. [38] pointed out that *O*-acetyl-arabino-4-*O*-methylglucuronoxylan was the main associated polysaccharide in Björkman LCC from gramineous *Arundo donax* Linn. Yuan et al. [39] reported that the acetylated 4-O-methylgluconoxylan was the main associated carbohydrate in poplar LCC. These results indicated that the structural properties of LCC are dependent on the plant species, resulting in different biorefinery processes, such as those influencing the efficiency of enzymatic saccharification and the interaction of lignin with cellulase [40].

**Figure 6:**
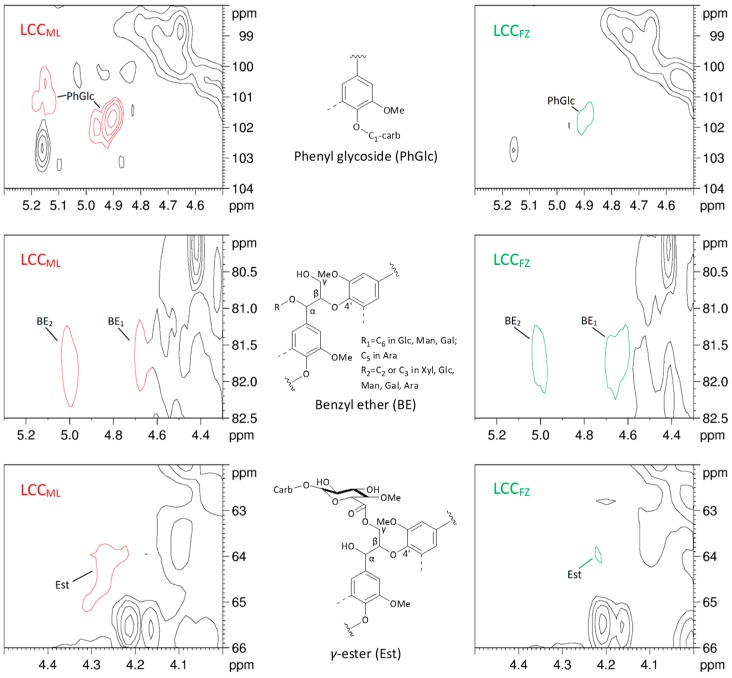
Partially amplified signals, PhGlc (*δ*_C_/*δ*_H_ 104–98/5.3–4.5), BE (*δ*_C_/*δ*_H_ 82.5–80/5.3–4.3), and Est (*δ*_C_/*δ*_H_ 66–62/4.5–4.0), of LCC_ML_ and LCC_FZ_ in the HSQC NMR spectra and the structure of the main LCC linkages.

It is believed that phenyl glycoside (PhGlc) bonds, ester (Est), and benzyl ether (BE) are the main types of LCC linkages [16,41]. As shown in Figure 6, the cross-peaks of PhGlc linkages could be observed in the region of *δ*_C_/*δ*_H_ 104.0–98.0/5.30–4.50. The C_1_-H_1_ and C_3_-H_3_ correlations for PhGlc linkages in the HSQC spectrum of LCC_ML_ were clearly observed at *δ*_C_/*δ*_H_ 100.8/5.15 and 101.5/4.90, respectively. However, only the C_3_-H_3_ correlations in the spectrum of LCC_FZ_ were detected. In addition, the C_2_-H_2_ correlations for PhGlc linkages were not detected in both HSQC spectra of LCC_ML_ and LCC_FZ_. It was reported that classic Björkman LCC preparation was preferable for the analysis of PhGlc linkages by comparing the HSQC spectra of Björkman LCC, LCC-AcOH, and enzymatic hydrolysis of Björkman LCC [38]. Therefore, the PhGlc linkages may be mainly present in Björkman LCC of ginkgo shells.

BE linkages could be detected in the region of *δ*_C_/*δ*_H_ 82.5–80.0/5.30–4.30. Researchers have pointed out that BE structure can be divided into two types: the linkages between the *α*-position of lignin and primary OH groups of carbohydrates (at C_6_ of Glucan, Galactan and Mannan; C_5_ of Arabinan) and linkages between the *α*-position of lignin and secondary OH groups of carbohydrates, mainly of the lignin-xylan type [42]. In the present study, the C*_α_*-H*_α_* correlations in the BE structure were found at *δ*_C_/*δ*_H_ 81.6/4.64 (BE_1_) and 81.4/5.04 (BE_2_). Cross-peaks from benzyl ester linkages in the region of 77.0–75.0/6.20–6.00 were not detected in the LCCs.

The correlations for Est linkages cross-linked at C*_γ_* were clearly observed at *δ*_C_/*δ*_H_ 66.0–62.0/4.50–4.00. However, the signals are easily overlapped with signals of lignin substructures in the HSQC spectra such as the FA and PCA [43]. In addition, the signals of *γ*-esters are also overlapped with signals of *γ*-acetyl lignin moieties (A′), especially in woody lignins. The 600 MHz spectrometer does not allow distinguishing between the *γ*-signals of LCC and acetyl *γ*-esters of lignin due to broad and overlapping signals in this area. A previous report has pointed out that a 950 MHz spectrometer with CryProbe™ can overcome this problem [41].

### 3.6. Quantification of Lignin Structure and LCC Linkages

**Figure 7:**
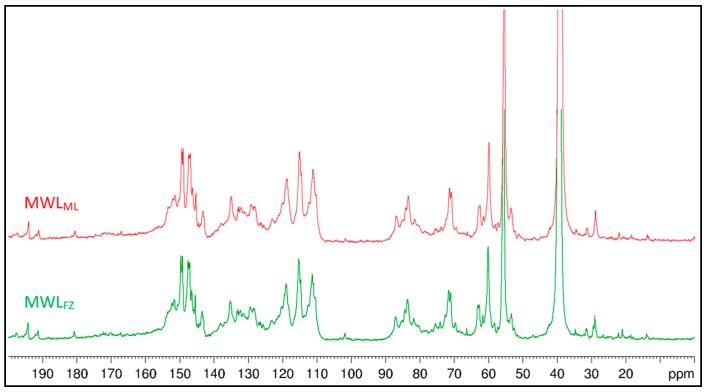
Quantitative ^13^C NMR spectra of MWL_ML_ and MWL_FZ_ from ginkgo shells.

Quantification of the lignin structure and LCC linkages is very important for providing comprehensive information about lignin architecture and reactivity, as well as for the biorefining process of lignocellulosic biomass. It will give guidance for the functional and chemical modification of lignin. ^13^C NMR spectroscopy is a reliable tool for lignin characterization. However, for the quantification of LCC linkages, appropriate internal references are needed to translate the relative values obtained from the integration of the HSQC spectra to absolute values. Due to the high stability of lignin aromatic rings, the number of specific structures per aromatic ring (/Ar), which is equivalent to one lignin monomeric unit, was often selected as the internal standard reference [19]. The values in the ^13^C NMR spectra (Figure 7) were related to the resonance of the aromatic carbons (163–103 ppm), which was set as 6.0. To quantify the LCC linkages, the values obtained from the integration of three clusters at 103–96 ppm, 90–78 ppm, and 65–58 ppm in the corresponding ^13^C NMR spectra were used. These clusters contain the signals of PhGlc, BE, and Est linkages, correspondingly. The amounts of LCC linkages were calculated per Ar as follows [19]:

Est = 2D_Est_/2D_65–58/5.0–2.5_ × ^13^C_65–58_/^13^C_163–103_ × 6.0 (1)

BE = 2D_BE_/2D_90–78/5.7–3.0_ × ^13^C_90–78_/^13^C_163–103_ × 6.0 (2)

PhGlc = 2D_PhGlc_/2D_103–96/5.5–3.8_ × ^13^C_103–96_/^13^C_163–103_ × 6.0 (3)

where Est, BE, and PhGlc are the amounts of *γ*-ester, benzyl ether, and phenyl glycoside linkages (per Ar); 2D_Est_, 2D_BE_, and 2D_PhGlc_ are the resonance of the signals of *γ*-ester, benzyl ether, and phenyl glycoside linkages in the 2D NMR spectra; 2D_65–58/5.0–2.5_, 2D_90–78/5.7–3.0_, and 2D_103–96/5.5–3.8_ are the total resonance of the corresponding clusters in the 2D NMR spectra; ^13^C_65–58_, ^13^C_90–78_, ^13^C_103–96_, and ^13^C_163–103_ are the resonance of the corresponding cluster in the ^13^C NMR spectra; and 6.0 is the amount of aromatic carbons per Ar. The results on the quantification of the lignin structure and LCC linkages are summarized in Table 4.

The amount of condensed structures (*β*-*β*′, *β*-1′ and *β*-5′) in MWL_ML_ (0.41/Ar) was lower than that in MWL_FZ_ (0.44/Ar), indicating that MWL_FZ_ had a higher condensation degree. Integration values of MWLs via 2D HSQC NMR also showed that the predominant aryl ether and condensed structure were *β*-O-4′ and *β*-5′ bonds, respectively. The condensed phenolic structure containing *β*-5′, 4-O-5′ and 5-5′ linkages is fairly resistant to oxidative degradation, even at higher temperatures [44]. Therefore, the high condensation degree may lead to ginkgo shells lignin having high recalcitrance in the process of biorefinery. However, the high condensation degree gives ginkgo shells lignin higher antioxidant activity than general softwoods lignin, which will play an important role in functional materials production. The amount of aliphatic and conjugated COOR groups was estimated to be 0.07/Ar and 0.02/Ar in MWL_ML_, and 0.09/Ar and 0.03/Ar in MWL_FZ_, correspondingly. The cluster at 90–77 ppm consists of various Alkyl-*O*-aryl and *α*-*O*-Alkyl moieties. The cluster at 77–65 ppm embodies moieties with *γ*-*O*-Alk ethers and secondary -OH groups. The high amount of Alkyl-*O*-moieties in MWLs from ginkgo shells indicated that the great mass of lignin units was etherified in the lignification process. The aromatic region of the lignin ^13^C NMR spectra can be classified into three broad categories: quaternary oxygenated (C_Ar-O_), nonoxygenated (C_Ar-C_), and methine (C_Ar-H_), which is important to quantify different condensed moieties such as 5-5′ and 4-O-5′ linkages of phenolic and etherified types.

The amount of BE linkages was 0.008/Ar in ginkgo shells, which was similar to that in Birch [41] but was lower than that in *Arundo donax* Linn [38], poplar MWL, and mild acidolysis lignin [39]. The amount of phenyl glycoside linkages in LCC_ML_ (0.035/Ar) was higher than that in LCC_FZ_ (0.027/Ar). However, the amount of *γ*-ester linkages in LCC_ML_ (0.026/Ar) was lower than that in LCC_FZ_ (0.039/Ar). These results suggest that the properties of LCCs depend on the plant species and the degree of lignification.

### 3.7. ^31^P NMR Spectra of MWLs

**Figure 8:**
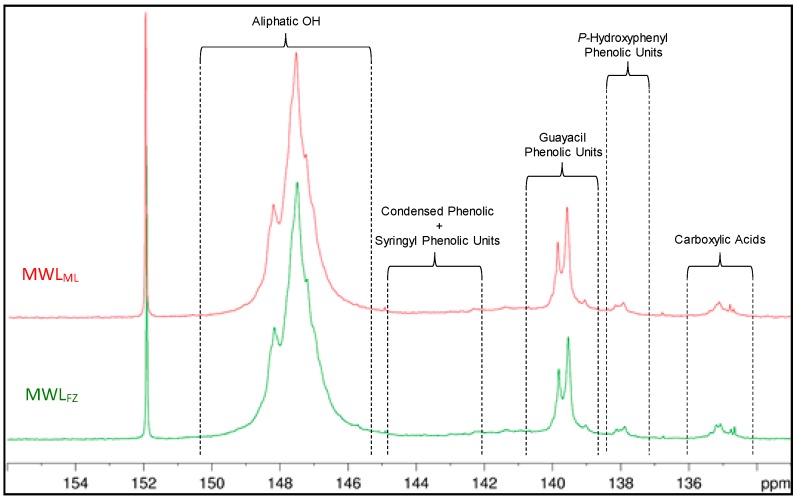
^31^P NMR spectra of MWL_ML_ and MWL_FZ_.

The content of hydroxyl groups can be determined by ^31^P NMR spectroscopy (see Figure 8), which offers important chemical reactivity and biological information. The results obtained from ^31^P NMR are included in Table 5. The phenolic hydroxyl was mainly derived from G units, with the amount of 0.78 mmol/g and 0.69 mmol/g for MWL_ML_ and MWL_FZ_, respectively, which is much lower than that in spruce (1.51 mmol/g), eucalyptus (2.09 mmol/g), and wheat straw (2.06 mmol/g) [15]. The results indicated that lignin in ginkgo shells has a high etherification and condensation degree. In consideration of the amount of *β*-O-4′ linkages (40%) in MWL_ML_ and MWL_FZ_, it is reasonable to deduce that the amount of *α*-O-4′ and 4-O-5′ bonds are the main condensed etherified structure. Although the low-level phenolic hydroxyl content in ginkgo shells lignin is beneficial to protect the fruit in ginkgo shells from pathogene and corruption, it reduces the application beyond its current roles, especially in biorefinery. The lower content of phenolic hydroxyl in MWL_FZ_ compared to that in MWL_ML_ implied the MWL_FZ_ had a higher condensation degree, which is line with the analysis of the ^1^H, ^13^C, and 2D HSQC NMR. However, the MWL_FZ_ had more carboxyl groups than MWL_ML_, suggesting that MWL_FZ_ may have a higher oxidation degree than MWL_ML_.

### 3.8. Nitrobenzene Oxidation

Nitrobenzene oxidation is a crucial supplement of NMR spectroscopy, which provides information on the aromatic rings and condensation degree of lignin. The yield and ratio of nitrobenzene oxidation products of ginkgo shells and LCCs are listed in Table 6. The lower products yield of FZ (1.79 mmol/g-lignin) compared to that of ML (1.64 mmol/g-lignin) suggested that lignin in FZ had a higher condensation degree than that in ML, being in line with the NMR analysis. As shown in Figure 2 and Table 4, the amount of *β*-5′ linkages was obviously higher than that of other condensed structures (*β*-1′, *β*-*β*′). Analysis of ^31^P NMR of MWLs also exhibited a high amount of *α*-O-4′ and 4-O-5′ structures in ginkgo shells lignin, which resulted in the nitrobenzene oxidation products yield of both ginkgo shells being much lower than that in general softwoods [45,46]. Tamai et al. [45] pointed out that the total yield of vanillin and vanillic acid of cedar (*Cryptomeria japonica*) under nitrobenzene oxidation was 1.95–1.99 mmol/g-lignin, which is about 17% and 26% higher than that in ML and FZ, respectively. The high condensation degree of ginkgo shells lignin may also explain why the MWL yield (17.5% and 15.3% for MWL_ML_ and MWL_FZ_, respectively) from ginkgo shells is low.

As shown in Table 6, the yield of H units in ginkgo shells was higher than that in Pine [46]. The structure of PCA in ginkgo shells is contributed to H units. Furthermore, *p*-coumaroyl-CoA 3-hydroxylase (C3H) is a monooxygenase that catalyzes the 3-hydroxylation of *p*-coumaroyl shikimate and *p*-coumaroyl quinate. C3H easily becomes a rate-limiting step in lignin biosynthesis and it is not surprising that an extreme reduction in C3H activity results in an increase of *p*-hydroxyphenyl monomers [47]. The inactivation of C3H further supports the involvement of hydroxycinnamic acid shikimate esters in the lignin biosynthetic pathway, causing the high content of H units in nitrobenzene oxidation products. The higher amount of acid soluble lignin in LCCs than that in raw materials (Table 1) implies that the lignin in LCCs has a lower molecular weight. Therefore, the condensation degree of lignin in LCCs was much lower than that in raw materials, causing the LCCs to have a higher nitrobenzene oxidation products yield than ginkgo shells.

## 4. Conclusions

The chemical composition, structural properties of lignin, and lignin-carbohydrate complex in ginkgo shells are rather different from those in general softwoods. Ginkgo shells analyzed in this work have a higher lignin and xylan content but less cellulose when compared with general softwoods. Isolated milled wood lignins are rich in guaiacyl units and have a high condensation and etherification degree. Although hardly detected in general softwoods lignin, Ferulates and *p*-coumarates are detected in ginkgo shells lignin, and the biosynthesis pathway of ferulates is rather different from that of *p*-coumarates. Aryl ether *β*-O-4′ substructures are the prominent linkages, followed by condensed *β*-5′, *α*-O-4′, and 4-O-5′ bonds. The molecular formula of MWL_ML_ and MWL_FZ_ is C_9_H_7.93_O_2.73_(OCH_3_)_0.81_ and C_9_H_7.87_O_2.76_(OCH_3_)_0.88_, respectively. MWL_ML_ has a higher phenolic hydroxyl content but with lower –OCH_3_ and carboxyl hydroxyl content than MWL_FZ_. *O*-acetylated *β*-d-xylopyranoside and *β*-d-mannopyranoside units are the main carbohydrates associated with lignin. LCC_ML_ has a higher phenyl glycoside, lower *γ*-ester, and similar benzyl ether content compared with LCC_FZ_. The higher content of lignin and the lignin-xylan complex, and the higher condensation degree of lignin in ginkgo shells compared with that in general softwoods, are considered to be the main reason for the high rigidity and antioxidant activity.

## Figures and Tables

**Table 1:** Chemical composition and yield of ginkgo shells, MWLs, and LCCs (%).

Samples ^1^	Carbohydrates			Lignin		Ash	Yield ^2^
	Glucan	Xylan	Arabinan + Mannan	Klason	Acid Soluble		
ML	21.3 ± 0.1	19.9 ± 0.7	1.9 ± 0.3	42.4 ± 0.2	0.7 ± 0.2	0.8 ± 0.1	-
FZ	20.0 ± 0.4	20.2 ± 0.2	1.5 ± 0.1	42.0 ± 0.2	0.7 ± 0.2	0.9 ± 0.1	-
MWL_ML_	0.2 ± 0.0	1.1 ± 0.0	0.3 ± 0.0	93.6 ± 1.5	3.9 ± 0.0	0.0	17.5
MWL_FZ_	0.9 ± 0.1	2.9 ± 0.2	0.4 ± 0.1	90.3 ± 1.8	3.9 ± 0.1	0.0	15.3
LCC_ML_	12.0 ± 0.7	17.7 ± 0.7	3.1 ± 0.5	24.5 ± 1.5	17.9 ± 0.2	1.4 ± 0.2	N.D.
LCC_FZ_	13.7 ± 0.1	17.8 ± 0.4	2.7 ± 0.1	24.1 ± 1.5	17.5 ± 0.1	1.6 ± 0.1	N.D.

^1^ The amount of benzene-ethanol extractives in ML and FZ was 5.7% and 8.1%, respectively; ^2^ N.D., Not Detected.

**Table 2:** Elemental analysis and distribution of molecular weight of MWL_ML_ and MWL_FZ_.

Samples	C	H	N	S	O	M_w_	M_n_	M_w_/M_n_
MWL_ML_	62.5	5.5	1.5	0.4	30.1	12,130	3800	3.19
MWL_FZ_	62.1	5.6	1.5	0.3	30.5	11,550	3930	2.94

**Table 3:** Assignment of the polysaccharide signals in the 2D HSQC NMR spectra of LCCs.

Label	*δ*_C_/*δ*_H_ (ppm)	Assignment
Est	65-62/4.5-4.0	C-H in *γ*-ester linkages
X_5_	62.6/3.40	C_5_-H_5_ in *β*-d-xylopyranoside
M2_2_	70.3/5.27	C_2_-H_2_ in 2-*O*-acetyl-*β*-d-mannopyranoside
X_2_	72.6/3.05	C_2_-H_2_ in *β*-d-xylopyranoside
X2_2_	72.8/4.43	C_2_-H_2_ in 2-*O*-acetyl-*β*-d-xylopyranoside
M3_3_	73.6/4.93	C_3_-H_3_ in 3-*O*-acetyl-*β*-d-mannopyranoside
X_3_	73.8/3.25	C_3_-H_3_ in *β*-d-xylopyranoside
X3_3_	73.9/4.61	C_3_-H_3_ in 3-*O*-acetyl-*β*-d-xylopyranoside
X_4_	75.4/3.53	C_4_-H_4_ in *β*-d-xylopyranoside
U_4_	81.1/3.10	C_4_-H_4_ in 4-*O*-methyl-*α*-d-glucuronic acid
BE_2_	81.4/5.04	C*_α_*-H*_α_* in benzyl ether (primary OH) linkages
BE_1_	81.6/4.64	C*_α_*-H*_α_* in benzyl ether (secondary OH) linkages
*α*X_1(R)_	91.9/4.89	C_1_-H_1_ in (1→4)-*α*-d-xylopyranoside (R)
*β*X_1(R)_	96.8/4.26	C_1_-H_1_ in (1→4)-*β*-d-xylopyranoside (R)
U_1_	97.0/5.07	C_1_-H_1_ in 4-*O*-methyl-*α*-d-glucuronic acid
X23_1_	98.3/4.72	C_1_-H_1_ in 2,3-*O*-acetyl-*β*-d-xylopyranoside
X2_1_	99.8/4.52	C_1_-H_1_ in 2-*O*-acetyl-*β*-d-xylopyranoside
X3_1_	101.3/4.28	C_1_-H_1_ in 3-*O*-acetyl-*β*-d-xylopyranoside
PhGlc_3_	101.5/4.90	C_3_-H_3_ in phenyl glycoside linkages
X_1_/Glc_1_	103.0/4.31	C_1_-H_1_ in *β*-d-xylopyranoside/*β*-d-glucopyranoside

**Table 4:** Lignin and LCC linkage characteristics from the integration of quantitative ^13^C and 2D HSQC NMR spectra.

Range (ppm)	Assignment	Amount	
		ML	FZ
Lignin characteristic ^1^			
199–196	C*_α_* = O except G′	0.07	0.05
196–193	C = O in C*_α_* = O/*β*-O-4′ (A′), F, J	0.08	0.07
193–190	Ar-CHO	0.07	0.07
182–179	C_4_ in F (*β*-1′)	0.03	0.03
175–168.5	aliphatic COOR	0.07	0.09
168.5–166	conjugated COOR	0.02	0.03
159–151	C*_α_* in J, C_3, 6_ in F, C_4_ in conjugated CO/COOR etherified units	0.29	0.25
144.5–142.5	C_3_ in B (*β*-*β*′)	0.20	0.25
57–54	–OCH_3_, C_1_ in F	1.05	1.08
54–52	C*_β_* in B and C (*β*-*β*′, *β*-5′)	0.18	0.16
Clusters ^1^			
163–142	aromatic C-O	1.85	1.79
142–125	aromatic C-C	1.52	1.56
125–102	aromatic C-H	2.62	2.67
90–58	Alk-*O*-	2.23	2.36
90–77	Alk-*O*-Ar, α-*O*-Alk	0.83	0.83
77–65	*γ*-*O*-Alk, secondary OH	0.68	0.75
Interunit linkages and structural units ^2^			
111.0/6.98	guaiacyl units (G)	99	99
128.0/7.20	*p*-hydroxyphenyl units (H)	1	1
71.2/4.74	*β*-O-4′ alkyl ether linkages (A′)	40	41
87.0/5.45	phenylcoumaran substructures (B)	12	14
84.9/4.63	resinol substructures (C)	3	4
83.3/4.83	dibenzodioxocin substructures (D)	1	2
81.4/5.03	spirodienone substructures (F)	<1	<1
LCC linkages ^3^			
104.0–98.0/5.30–4.50	PhGlc	0.035	0.027
82.5–80.0/5.30–4.30	BE	0.008	0.008
66.0–62.0/4.50–4.00	Est	0.026	0.039

^1^ Quantified according to the method described by Capanema et al. [13]; results expressed per Ar; ^2^ Molar percentages (H + G = 100); Interunit linkages molar contents as percentages of lignin content; ^3^ The sum of LCC *γ*-ester and *γ*-acylated *β*-O-4′ aryl ether substructures (A′); results expressed per Ar.

**Table 5:** Compositional analysis from ^31^P NMR spectra of MWLs (mmol/g).

Samples	Aliphatic OH	Phenolic OH	C + S	G	H	COOH
MWL_ML_	4.44	1.38	0.42	0.78	0.18	0.16
MWL_FZ_	4.30	1.23	0.39	0.69	0.15	0.18

**Table 6:** The yield and ratio of nitrobenzene oxidation products of ginkgo shells and LCCs.

Samples	Yield (mmol/g-lignin)				V/S/H ^1^
	V	S	H	Total	
ML	1.65 ± 0.02	0.02 ± 0.00	0.11 ± 0.01	1.79 ± 0.01	92/1/7
FZ	1.47 ± 0.05	0.02 ± 0.00	0.15 ± 0.01	1.64 ± 0.02	90/1/9
LCC_ML_	2.84 ± 0.09	0.02 ± 0.00	0.14 ± 0.00	2.25 ± 0.03	95/1/4
LCC_FZ_	2.14 ± 0.00	0.02 ± 0.00	0.10 ± 0.00	3.00 ± 0.00	95/1/4

^1^ V = vanillin + vanillic acid; S = syringaldehyde + syringic acid; H = *p*-hydroxybenzaldehyde + *p*-hydroxybenzoic acid.
